# Subfoveal choroidal thickness changes in carotid cavernous fistula following spontaneous resolution

**DOI:** 10.1186/s12886-016-0240-2

**Published:** 2016-05-26

**Authors:** Amanda Rey, Lorena Castillo, Agnieszka Dyrda, Xavier Maseras, Ignasi Jürgens

**Affiliations:** Institut Catala de Retina (ICR), Ganduxer, 117, 08022 Barcelona, Spain

**Keywords:** Choroidal thickness, Optical coherence tomography, Carotid cavernous fistula, Serous retinal detachment

## Abstract

**Background:**

We report the enhanced depth imaging optical coherence tomography (EDI-OCT) characteristics and variations in a patient with subretinal fluid secondary to a carotid cavernous fistula.

**Case presentation:**

A 59-year-old man presented with blurred vision in his right eye. Venous congestion of the epiescleral and retinal vessels were observed. EDI-OCT disclosed macular subretinal fluid with an increase of choroidal thickness up to 341 μm. Brain and orbital computerized tomography showed an enlarged right superior ophthalmic vein. Orbital magnetic resonance imaging and angiography disclosed a decrease in blood flow, an indirect sign of carotid cavernous fistula. After a 3 months follow-up, spontaneous closure of the fistula occurred. Both the dilation of the conjunctiva and retinal veins improved. EDI-OCT showed resolution of the subfoveal fluid and a reduction of the subfoveal choroidal thickness to 271 μm after a 3 months follow-up and 168 μm after a 8 months follow-up.

**Conclusion:**

Serous retinal detachment has been described as a rare complication of carotid cavernous fistula. In our patient, EDI-OCT examinations revealed a thicker choroidal thickness when subretinal fluid was present as compared to that observed in the contralateral eye or after subretinal fluid resolution.

## Background

Carotid cavernous fistula (CCF) is an abnormal shunt between the carotid arterial system and the cavernous sinus, which typically presents with oculo-orbital venous congestive features [1]. We report the enhanced depth imaging optical coherence tomography (EDI-OCT; Spectralis; Heidelberg Engineering, Heidelberg, Germany) characteristics and variations in a patient with subretinal fluid secondary to a carotid cavernous fistula.

## Case presentation

**Fig. 1:**
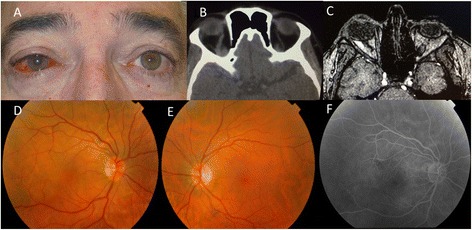
**a** Initial photograph showing tortuous and dilated episcleral vessels of the patient’s right eye. **b** CT shows an enlarged right superior ophthalmic vein. **c** MRA suggested a reduction of blood flow, which led to the suspicion of cavernous sinus-dural arteriovenous fistula. **d** Fundus photography of the right eye with venous congestion of the retinal veins and macular subretinal fluid. **e** The normal left eye. **f** Fluorescein angiography without clear leakage in the area of the serous detachment

**Fig. 2:**
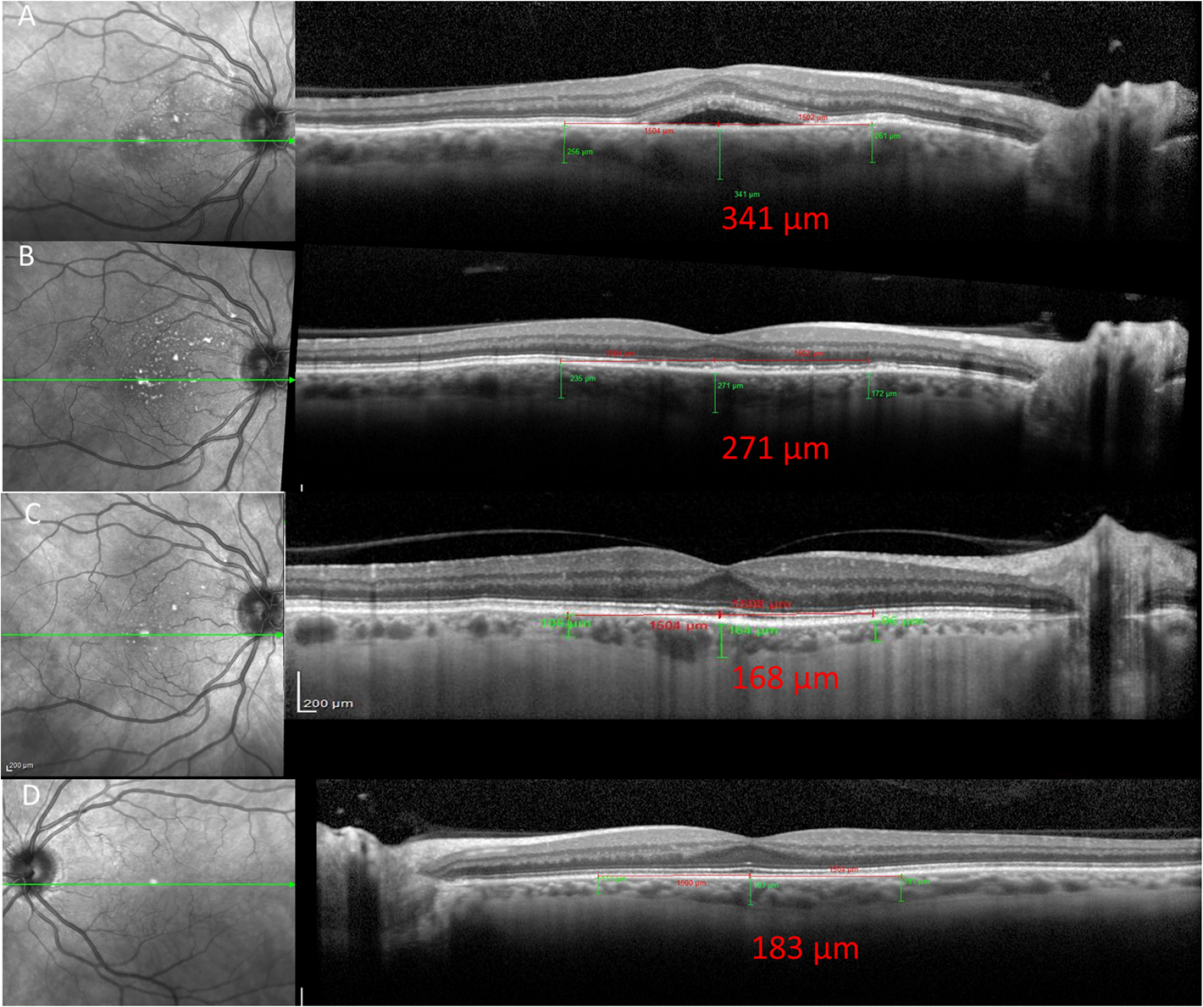
**a** Macular subretinal fluid on OCT with a choroidal thickness of 341 μm in his right eye. **b** Resolution of the subfoveal fluid and a reduction of the subfoveal choroidal thickness to 271 μm after spontaneous closure of the fistula. **c** Reduction of the subfoveal choroidal thickness to 168 μm after a 8 months follow-up. **d** The normal left eye with a choroidal thickness of 183 μm

A 59-year-old man presented with a 1-month history of redness and blurred vision in his right eye. Best-corrected visual acuity in his right and left eye was 20/30 and 20/20, respectively. The episcleral vessels were tortuous and dilated in his right eye (Fig. 1a). Right abducens cranial nerve palsy was detected. Intraocular pressure was 18 mmHg in his right eye and 14 mmHg in his left eye. Hertel exophthalmometry was 22 mm on the right and 18 mm on the left. Venous congestion of the retinal veins and macular subretinal fluid were observed in his right eye (Fig. 1d). EDI-OCT disclosed macular subretinal fluid with an increase of choroidal thickness up to 341 μm in his right eye (Fig. 2a). His left eye was normal (Fig. 1e) with a choroidal thickness of 183 μm (Fig. 2d). Fluorescein angiography did not show a clear leakage in the area of the serous detachment (Fig. 1f).

Laboratory tests, which included thyroid profiles, were all within the normal ranges. Brain and orbital computerized tomography (CT) showed an enlarged right superior ophthalmic vein (Fig. 1b). Magnetic resonance imaging (MRI) and angiography (MRA) suggested a decrease in blood flow, an indirect sign of carotid cavernous fistula (Fig. 1c).

After a 3 months follow-up, spontaneous closure of the fistula occurred. Both the dilation of the conjunctiva and retinal veins improved. Best-corrected visual acuity in his right eye was 20/20. EDI-OCT showed resolution of the subfoveal fluid and a reduction of the subfoveal choroidal thickness to 271 μm after a 3 months follow-up (Fig. 2b) and 168 μm after a 8 months follow-up (Fig. 2c).

## Conclusion

The choroid is the vascular layer that supplies the outer retina and is involved in the pathogenesis of several ocular conditions including choroidal tumors, age related macular degeneration, central serous chorioretinopathy, diabetic retinopathy, and uveitis. EDI-OCT is a noninvasive technique that is used to evaluate choroidal thickness and morphology in these diseases [2]. Shinohara et al. [3], have already reported that the relationship between an increased choroidal thickness by EDI-OCT and choroidal congestion in CCF by using fluorescein angiography, laser speckle flowgraphy and brain angiography before and after embolization of the CCF, however, serous retinal detachment was not present.

Serous retinal detachment has been described as a rare complication of carotid cavernous fistula. Choi et al.​ [4] have already indicated that they observed serious retinal detachment secondary to CCF using Stratus OCT and the complication disappeared after the treatment for CCF, similarly to Garg et al. [5], however they did not evaluate choroidal thickness during the presence of serous retinal detachment.

In our patient, EDI-OCT examinations revealed a thicker choroidal thickness when subretinal fluid was present as compared to that observed in the contralateral eye or after subretinal fluid resolution. These findings are similar to those observed in central serous choroidopathy [6]. Its pathogenesis remains unknown. It is thought that arterialization of the orbital veins causes venous stasis, congestion of the choriocapillaris which then leads to hypoxia and subsequent impairment of retinal pigment epithelial cell function. Dysfunction of the choriocapillaris and retinal pigment epithelium has been shown to lead to serous retinal detachment. This congestion may be followed-up by measuring non-invasively the choroidal thickness. On normalization of orbital venous outflow, the choriocapillaris and retinal pigment epithelial function returns to normal, the serous retinal detachment resolves, and the choroidal thickness returns to normal.

To our knowledge, this is the first reported case of serous macular detachment secondary to carotid cavernous fistula in which EDI-OCT shows choroidal thickness changes after spontaneous resolution. The technique provides a detailed objective in vivo visualization of the choroid that can be used to monitor disease activity.

## Abbreviations

CCF, carotid cavernous fistula; CT, computerized tomography; EDI-OCT, enhanced depth imaging optical coherence tomography; MRA, magnetic resonance angiography; MRI, magnetic resonance imaging.
